# A National, Semantic-Driven, Three-Pillar Strategy to Enable Health Data Secondary Usage Interoperability for Research Within the Swiss Personalized Health Network: Methodological Study

**DOI:** 10.2196/27591

**Published:** 2021-06-24

**Authors:** Christophe Gaudet-Blavignac, Jean Louis Raisaro, Vasundra Touré, Sabine Österle, Katrin Crameri, Christian Lovis

**Affiliations:** 1 Division of Medical Information Sciences Geneva University Hospitals Geneva Switzerland; 2 Department of Radiology and Medical Informatics University of Geneva Geneva Switzerland; 3 Data Science Group Division of Information Systems Lausanne University Hospital Lausanne Switzerland; 4 Precision Medicine Unit Department of Laboratories Lausanne University Hospital Lausanne Switzerland; 5 Personalized Health Informatics Group SIB Swiss Institute of Bioinformatics Basel Switzerland

**Keywords:** interoperability, clinical data reuse, personalized medicine

## Abstract

**Background:**

Interoperability is a well-known challenge in medical informatics. Current trends in interoperability have moved from a data model technocentric approach to sustainable semantics, formal descriptive languages, and processes. Despite many initiatives and investments for decades, the interoperability challenge remains crucial. The need for data sharing for most purposes ranging from patient care to secondary uses, such as public health, research, and quality assessment, faces unmet problems.

**Objective:**

This work was performed in the context of a large Swiss Federal initiative aiming at building a national infrastructure for reusing consented data acquired in the health care and research system to enable research in the field of personalized medicine in Switzerland. The initiative is the Swiss Personalized Health Network (SPHN). This initiative is providing funding to foster use and exchange of health-related data for research. As part of the initiative, a national strategy to enable a semantically interoperable clinical data landscape was developed and implemented.

**Methods:**

A deep analysis of various approaches to address interoperability was performed at the start, including large frameworks in health care, such as Health Level Seven (HL7) and Integrating Healthcare Enterprise (IHE), and in several domains, such as regulatory agencies (eg, Clinical Data Interchange Standards Consortium [CDISC]) and research communities (eg, Observational Medical Outcome Partnership [OMOP]), to identify bottlenecks and assess sustainability. Based on this research, a strategy composed of three pillars was designed. It has strong multidimensional semantics, descriptive formal language for exchanges, and as many data models as needed to comply with the needs of various communities.

**Results:**

This strategy has been implemented stepwise in Switzerland since the middle of 2019 and has been adopted by all university hospitals and high research organizations. The initiative is coordinated by a central organization, the SPHN Data Coordination Center of the SIB Swiss Institute of Bioinformatics. The semantics is mapped by domain experts on various existing standards, such as Systematized Nomenclature of Medicine Clinical Terms (SNOMED CT), Logical Observation Identifiers Names and Codes (LOINC), and International Classification of Diseases (ICD). The resource description framework (RDF) is used for storing and transporting data, and to integrate information from different sources and standards. Data transformers based on SPARQL query language are implemented to convert RDF representations to the numerous data models required by the research community or bridge with other systems, such as electronic case report forms.

**Conclusions:**

The SPHN strategy successfully implemented existing standards in a pragmatic and applicable way. It did not try to build any new standards but used existing ones in a nondogmatic way. It has now been funded for another 4 years, bringing the Swiss landscape into a new dimension to support research in the field of personalized medicine and large interoperable clinical data.

## Introduction

### Background

Interoperability is a well-known challenge in medical informatics and is one of the main obstacles preventing data-driven medicine from realizing its full potential. Efforts to classify and express meaning in health care are as old as the International Classification of Diseases (ICD) [1]. Organizations, such as Health Level Seven International (established in 1987) [2] and SNOMED International, which maintains and releases the Systematized Nomenclature of Medicine Clinical Terms (SNOMED CT) [3], are dedicated to promoting interoperability in health care. Moreover, multiple national and international programs are seeking to promote interoperability. Examples of major initiatives designed to tackle the interoperability challenge in health care include the Meaningful Use program under the Health Information Technology for Economic and Clinical Health Act [4] in the United States and the Integrating the Healthcare Enterprise initiative [5], with more than 175 member organizations worldwide.

### Semantic Interoperability

Semantic interoperability usually involves controlled vocabularies. In the medical field, the equivalent is part of the culture, but is named differently, such as scales, scores, and classifications. These involve organization of medical knowledge into a finite set of classes. They are used in daily practice to evaluate, describe, and prognose many situations or conditions. For example, medical scales or scores are narrow-scope classifications used in everyday medical practice. The Glasgow Coma Scale [6] and the Apgar score [7] are examples to describe the level of consciousness of patients and the health of newborns, respectively. In some cases, there are several of them for a specific condition with different perspectives, such as for heart failure [8]. Clinicians commonly use dozens of scores and scales in their daily practice, and there are numerous applications that combine and facilitate their use [9,10].

More extensive medical classifications, such as the 10th revision of the International Classification of Diseases (ICD-10) [11] and the Logical Observation Identifiers Names and Codes (LOINC) [12], are large systems that attempt to organize broader areas of medical knowledge, such as diseases and causes of death (ICD-10), or health measurements, observations, and documents (LOINC).

They can be articulated into larger representations (meta-organizations) that consolidate several classifications, ontologies, terminologies, etc. The Unified Medical Language System (UMLS) Metathesaurus [13,14], for example, combines several classifications having different purposes, such as diagnosis encoding and literature indexing. SNOMED CT is another example, which combines 19 top-level hierarchies into one representation.

Specific classifications are characterized by a partitioning of the knowledge represented according to a specific purpose, usually the intention for which the classification has been designed. Thus, SNOMED CT is historically dedicated to pathology and was extended later with clinical codes. ICD-9 and 10 are well adapted to represent diagnosis and morbidity causes, while LOINC is mostly used to represent laboratory analytical and preanalytical characteristics. Drugs are often handled using Global Trade Item Number (GTIN) for logistical needs and Anatomical Therapeutic Chemical (ATC) classifications for order entry decision support [15,16], while adverse drug reactions are reported in MedDRA [17].

#### Challenges for Semantic Interoperability

As a result of having specific classifications well designed for specific purposes, they are usually not well adapted to express other types of knowledge or different organizations (partitioning) of that knowledge. SNOMED CT is able to represent almost any pathological test result and has been used to represent free text, but it fails to express some types of concrete values [18,19]. ICD-10 can be used to assign a code to any disease, but its mono-hierarchical structure prevents meaningful information reuse (eg, it is not possible to easily extract all codes representing infectious diseases). Finally, GTIN identifies commercial drug products, but it does not efficiently represent active substances, while ATC expresses only substances, but not the products. Classifications are tools used to represent the meaning of the data, but they always carry an intent, and none can be used for every purpose.

### Data Organization

Data are usually organized with data models, and the first and most simple is the text or tabular file that is still widely used, notably in clinical research settings. The serialization of data in comma-separated value (CSV) files can be expanded into more complex representations. Data models structure the data into entities and relationships that fit a given purpose. These have existed in health care for a long time, and some of them are widely used. Health Level 7 (HL7) version 2, which is the most widely implemented standard for health care in the world [20], is linked to the Reference Information Model (RIM), a data model designed to be the backbone of HL7, with the following three main classes: Act (representing something that has happened or will happen), Entity (any living or nonliving thing), and Role (a competency expressed by an Entity). These three classes will then be used to build an event using a “connector” named “Participation” that allows building of complex nested structures [21]. Finally, as for controlled vocabularies, data models can be articulated in meta-models, such as the bridge recently created between the Observational Medical Outcomes Partnership (OMOP) and the Informatics for Integrating Biology and the Bedside (i2b2) [22] data models.

#### Challenges of Data Interoperability

The structure of each data model depends on the goal of the standard and on the community that will use the data. For example, the RIM was primarily targeted at electronic health record (EHR) interoperability, while the Common Data Model of OMOP specifically targeted clinical research [23]. The data model of i2b2 [24] is designed to integrate genetic and phenotypic data, while the Clinical Data Interchange Standards Consortium (CDISC) operational data model [25] is required for drug regulatory constraints by the United States Food and Drug Administration. The openEHR project is built around another paradigm and is composed of archetypes that are small domain models aimed at providing a specific piece of information. The definition of archetypes and templates of archetypes are very flexible and can solve numerous interoperability challenges; however, it still requires adopting the reference model for the storage of data [26,27]. The design of these models is based on specific goals, and there is no one-size-fits-all data model that can serve every purpose.

### The Swiss Personalized Health Network

The Swiss Personalized Health Network (SPHN) aims to leverage research in the field of personalized health in Switzerland by building a nationally coordinated infrastructure network that supports exchange and reuse of health-related data produced by the health care system and in biomedical and clinical research settings [28]. This national initiative was launched in 2017, with funding of up to CHF 137 million (US $153 million) assured until 2024 [29,30]. In essence, the goal of the SPHN is to connect the Swiss health care system, the research community, regulatory agencies, and eventually industrial partners involved in personalized medicine. Consequently, the SPHN is at the interface between three communities and must overcome the multiple challenges of exchanging data in a secure, interoperable, and meaningful manner.

### Objectives

The challenges of interoperability described above have been the focus of active research in recent decades. Every year, new standards appear with the goal of addressing the remaining challenges. Interestingly, each of these new standards solves some problems but also generates new ones.

As opposed to conventional approaches, which are aimed at mapping data to one common standard and are in practice only effective for specific use cases, our interoperability strategy uses existing standards in a purpose-specific and complementary manner without depending on any particular one, thus providing great flexibility and sustainability. As such, it enables data interoperability between various communities, each of which has different needs or follows different requirements with regard to the type of data model to be used.

### Vocabulary

Interoperability is by essence an interdisciplinary process. Therefore, the vocabulary used to describe its components can vary. This section aims to define the words used in this work and their meaning. *Data model* is an abstract model that organizes elements of data in structures. *Data model-independent* is used to describe a system that does not depend on a predefined data model. *Encoding* is the action of expressing something with a specific coding system. For example, encoding a concept into a terminology means linking this concept to the elements of the targeted terminology that adequately represent it. *Interoperability* is the ability of two different entities to connect, share, understand, and use data in their processes. *Semantics* is the encoding of meaning into one or more knowledge representations (KRs). *Knowledge representation* is organization of knowledge into a list of elements, such as controlled vocabularies, terminologies, classifications, taxonomies, ontologies, thesauri, and coding systems.

## Methods

### Overview

**Figure 1:**
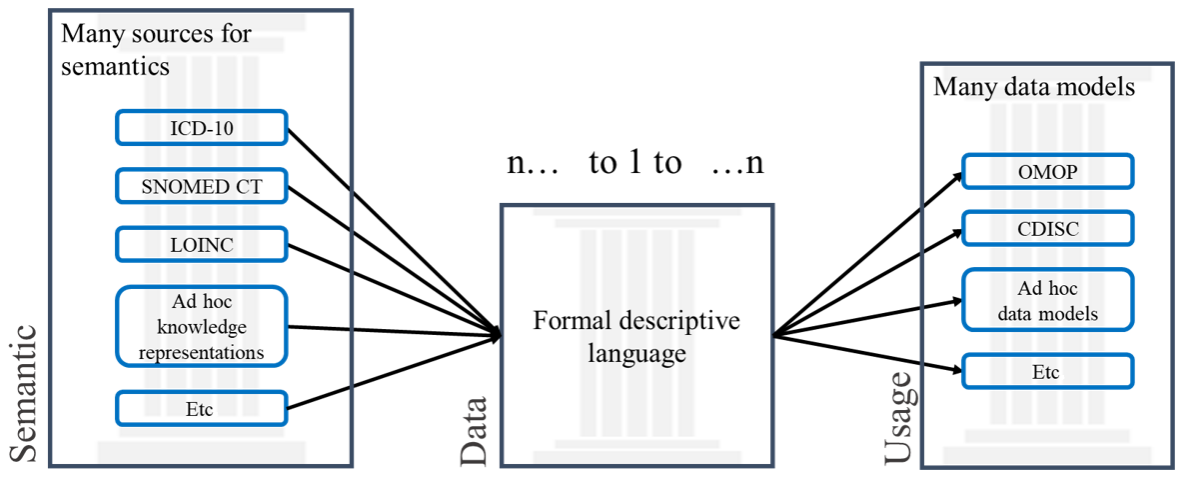
The three pillars of the proposed data interoperability strategy. CDISC: Clinical Data Interchange Standards Consortium; Etc: et cetera; ICD-10: International Classification of Diseases; LOINC: Logical Observation Identifiers Names and Codes; OMOP: Observational Medical Outcomes Partnership; SNOMED CT: Systematized Nomenclature of Medicine Clinical Terms.

Based on the lessons learned from previous attempts, this work addresses the interoperability challenge adopting a semantic-driven data model–independent framework based on the following three pillars (Figure 1):

1. A multidimensional encoding of the concepts. Only the required concepts (variables) are encoded in any KR system. This decision is completely agnostic, so that several international standards can be used at any time, according to the needs.
2. Resource description framework (RDF)-based storage and transport of the instances of these concepts when used to express clinical data. The RDF is well suited for a federated national exchange format. As it is a formal descriptive language, it is very scalable to any future needs not yet known.
3. Conversion of the RDF to any target data model that is needed for a specific research community or usage, according to the needs of the users.

This ends up with the first two pillars being completely data model independent. Only at the third pillar will the data be available in any required model, such as CDISC and OMOP, according to needs. We thus considered this strategy “semantic agnostic” and “model independent.”

This strategy is being implemented stepwise since January 2019. This paper focuses on the strategy. The deployment and societal challenges will be discussed in a further publication.

### Integrative and Usability-Focused Semantic Approach

As stated in the Introduction, it is illusory to believe that different communities will adopt a single standard for the sake of mutual compatibility. Therefore, this strategy does not enforce a specific KR to express meaning. The goal is to enable the use of an adequate KR, based on the purpose and context of use, without imposing any specific one. However, the presence of a semantic definition of the data is crucial and must be the central axis of the strategy.

The first pillar of our approach consists, therefore, of developing a semantic framework comprising a set of concept definitions relying on existing KRs or new ones if needed. The concept definition must be adapted to the granularity required by the use case. Each concept can be encoded into as many KRs as required. For instance, the concept “Heart Rate” can include encoding into SNOMED CT and LOINC. The power of representation and usability is prioritized over conceptualization. It is thus possible to express the meaning of the data without enforcing a specific KR. Finally, instantiations of the concepts can use an adequate KR, depending on the context. Axioms of the first pillar are summarized in Textbox 1.

**Axioms of the first pillar of the strategy.**

**Axioms**

- Framework composed of a set of concept definitions.
- Semantic encoding using a knowledge representation.
- Multiencoding of a concept in several knowledge representations allowed.
- Selection of concepts defined by use cases.
- Combination and extension of concepts allowed.

### Descriptive Formalism for Transfer and Storage

Transport and storage of information are essentially the same. Since transport is a “moving storage” and storage is a “nonmoving transport,” they can be regarded as a single challenge. The data and concept landscapes in health care are constantly evolving with new elements to be exchanged. To best answer this need for sustainability, scalability, and plasticity, the strategy is based on the use of a descriptive formalism (eg, the RDF, the Arden syntax, and the Web Ontology Language [OWL]) [31-33]. These languages offer flexible storage and transport of information (be it data, semantics, processes, or rules). This differs from a data model–based approach, as it does not constrain data to fit a specific format but only describes the data and its semantics in an intuitive and unconstrained way as it is collected at the source. Our approach allows the use of different formalisms when needed. For example, RDF can be used to store and transport the data, and the Arden syntax can be used to describe rules, such as automatic alert and clinical decision support systems [32]. Similarly, other formalisms can be used for other types of information and purposes (eg, Guidelines Interchange Format for guidelines [34] and Java Business Process Model for workflows [35]). Textbox 2 summarizes the approach for the second pillar.

**Axioms of the second pillar of the strategy.**

**Axioms**

- Common approach for storage and transport.
- No a priori definition of a data model.
- Use of descriptive formalisms to describe the data encoded in the first pillar.
- Choice of the formalism depending on the use case.

### Purpose-Specific Transformation to Data Models

The final building block of our strategy is the transformation of data from a flexible representation, based on formal descriptive languages, to a more rigid but application-oriented representation, such as relational data models. The goal is to provide a way of efficiently sharing data between different communities used to working with their own data models. As mentioned above, no common data model can be adopted by all communities, and mappings across data models are often partial because of incompatible information representations.

As a result of the first and second pillars, it is possible to create ad hoc conversions based on users’ needs. In particular, the use of a data model–independent formalism to store data enables the implementation of one-to-many mappings to any target data model. For example, existing work has already proposed the transformation of RDF resources into customized relational data models [36] or standard common data models, such as i2b2 [37,38] and OMOP [39]. This approach addresses the complexity of the current many-to-many mappings and will enable the sharing of data with any community, provided that the mapping is done while keeping the data unchanged. Textbox 3 summarizes the approach.

**Axioms of the third pillar of the strategy.**

**Axioms**

- Ad hoc conversions from the descriptive formalism of the second pillar to data models.
- Building of a reusable one-to-many mapping catalog.
- Selection of the targeted data models based on use cases.

## Results

### The SPHN

The proposed interoperability strategy was implemented to serve the data-sharing needs of the SPHN. The projects supported are all large multicentric projects, multihospitals, multiresearch centers, and data-driven research related to personalized medicine [28]. They vary in terms of not only methodology and research questions, but also the clinical data concepts requested from the data providers involved. The projects are designed to generalize the use of the Swiss General Consent, improve clinical data management systems on care providers, build a national data interoperability landscape for research, and leverage research organizations.

The defined approach was implemented by every university hospital and high research organization of Switzerland as the national standard for sharing clinical data. Twelve driver projects were funded and used the approach for their data needs.

### Organization

In the implementation of the first pillar, a semantic framework has been built and maintained by the SPHN Data Coordination Center (DCC). The DCC is the central hub for data interoperability in the SPHN and part of the SIB Swiss Institute for Bioinformatics. Its mandate is to coordinate the development of the specification of the structure and semantics of the SPHN data set, which describes the type of data that is available and potentially shareable within the network (hereafter referred to as the SPHN semantic data set). A full description of the DCC is available on the SPHN website [40].

### First Pillar

**Figure 2:**
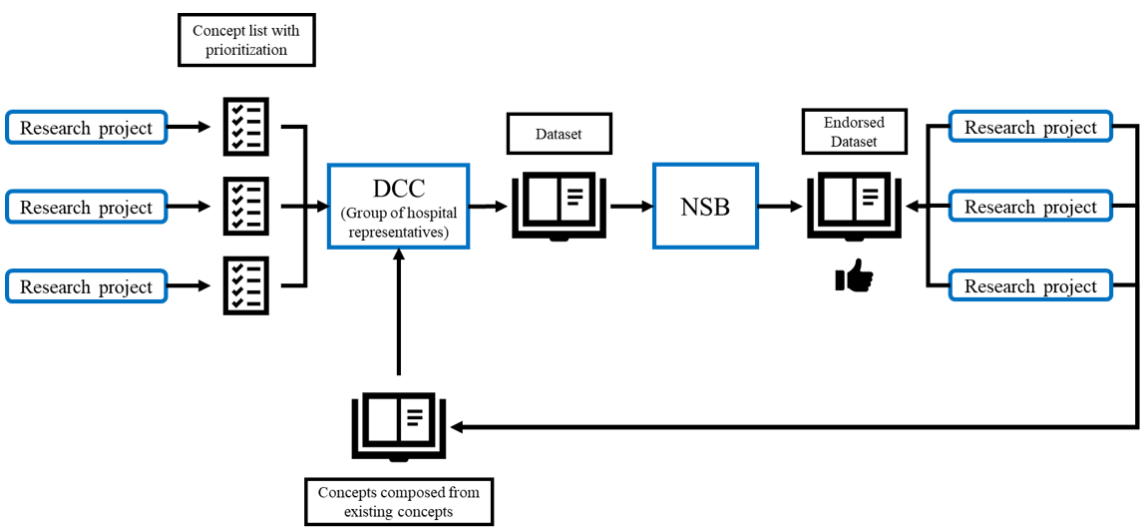
Flowchart of the validation process. DCC: Data Coordination Center; NSB: National Steering Board.

The content of the SPHN semantic data set is defined by leveraging domain knowledge from the Swiss clinical research community. Every research project provides the list of variables they need to the group in charge of aligning the semantics. This group includes domain experts and clinical semantics specialists. This SPHN semantic data set is periodically reviewed and extended according to experience obtained in projects by extracting and using the data and, of course, the new needs of research projects. There is a validation process that ends up in the publication of a new release of the core list of concepts endorsed by the SPHN National Steering Board (NSB). After official release, the new concepts are used by university hospitals for interoperable data exchange. The steps involved in this process are shown schematically in Figure 2. The complete structure of the SPHN is beyond the scope of this article and openly available in published reports [30].

The concept list is evolving, such that each element contains, in addition to semantics, management metadata, such as unique ID, a name, a description, and several fields for versioning. All data transfer for SPHN projects should comply with these concepts once enforced by the NSB. Examples of the encoding of these concepts with SNOMED CT and LOINC are shown in Table 1, in which the code is linked to the row where relevant and applicable. As more use cases arise, new encodings can be created.

The DCC has the task of exploring common international KR when validating new concepts, so as to select the most appropriate one. A KR for a concept is chosen taking into consideration not only its capacity to represent the concept correctly and unambiguously but also the ability of hospitals to comply with it and the research project to use it. Currently, more than 300 concepts are being used, which can describe demographics, laboratory analysis and results, drugs and prescriptions, clinical and physiological variables, etc [41].

**Table 1:** Examples of encoded concepts used to describe a temperature measurement.

Concept name	SNOMED CT^a^	LOINC^b^
Temperature	386725007 |Body temperature (observable entity)|	8310-5 Body temperature
Unit	767524001 |Unit of measure (qualifier value)|	N/A^c^
Body site	123037004 |Body structure (body structure)|	39111-0 Body site

^a^SNOMED CT: Systematized Nomenclature of Medicine Clinical Terms.

^b^LOINC: Logical Observation Identifiers Names and Codes.

^c^N/A: not applicable.

### Second Pillar

The data storage and transport step of the SPHN was implemented using RDF as proposed by the World Wide Web Consortium [42]. RDF allows to map instances of real data originating from a clinical database with the conceptual framework defined in the first pillar. The RDF allows to build a labeled directed multigraph, where nodes and edges are identified by uniform resource identifiers. The basic entity in the RDF graph is known as a “triple” and is composed of a subject, a predicate, and an object. Several triples compose a graph. Since the RDF does not depend on a specific semantic standard, it allows for the use of different ontologies and value sets, as required by the strategy. The reasons for choosing RDF technologies are summarized in Textbox 4 [43-47].

**Reasons for choosing the resource description framework.**

**Reasons**

- Flexibility to represent complex knowledge with simple statements (ie, triples of information).
- Scalability to other fields (eg, the resource description framework [RDF] has been adopted by systems biology and molecular biology for specific data representation [43,44]).
- Advanced query system (ie, with the SPARQL language).
- Existing tools in a rich community to create, maintain, validate, explore, and visualize RDF representation (eg, Protégé and WebVOWL [45-47]).

A set of rules and conventions has been defined to guide the creation of an SPHN RDF schema, that is, how RDF classes and properties required to generate instances (RDF resources) for storing hospitals’ data should be created [48,49]. Particularly, such rules stipulate (1) how concepts defined in the SPHN semantic data set should be converted into RDF classes or RDF properties and (2) how concepts that are not semantically linked to each other by composition should be linked to encapsulate contextual information provided at the time of data capture.

Swiss hospitals’ clinical research data warehouses are primarily based on relational database management systems. To transform data from a relational model representation into a graph representation based on RDF, extract, transform, and load (ETL) pipelines have been implemented by data providers’ informatics teams. They typically include an RDF transformer step where raw data from the EHR is converted and loaded into a triple store. Then, data can be extracted and serialized into RDF files for each specific project.

### Third Pillar

Converters are used to transform the RDF data into purpose-specific data models, serializing the RDF data into other common formats such as XML, JSON, JSON-LD, and TSV/CSV. For example, SPARQL queries have been implemented to convert data into flat files that can be processed by research-enabling software or machine learning pipelines [49].

## Discussion

### Overview

While the proposed data interoperability strategy offers a number of advantages in terms of flexibility and extensibility over more conventional approaches based on common data models, several challenges had to be addressed to allow effective implementation.

### Granularity Challenges

Finding the right representation for a concept is not trivial. Data can be represented in many ways (eg, “arm circumference” defined as a concept or a “circumference” concept connected with a “body site” concept taking the value “arm”), and agreeing on a common way to represent data is a challenging process. While both of these representations may be correct, interoperability is not always ensured if both are used, even though an international KR is used. This difference in the level of granularity also influences the way the user can query the data. When only one level of granularity is used in a specific data set, querying for relevant information is trivial. The user simply queries for the data of interest using the relevant defined concepts. However, if the data set comes from two different sources with different levels of granularity for the same type of information, either the querying needs to be adapted so that it can recognize both patterns or mapping must be performed beforehand to ensure that the results obtained are complete. Within the SPHN community, the granularity challenge has been addressed in the following two complementary ways: (1) when possible, a specific level is agreed by consensus and (2) in all other situations, all levels are encoded using a KR (for example SNOMED CT), allowing to query at different levels of granularity.

### Different Needs

Defining a common concept for different use cases proved to be complex when creating the semantic framework. Depending on the project, needs may vary widely. For example, one project may require the temperature of a patient, without any information on the site or the device used to measure it, while another project may require the exact device and site for the temperature. This problem is addressed by representing the meaning strongly, therefore allowing the different concepts to be represented. Thus, it is possible to express temperature and many additional (present and future) concepts, and associate them freely. This is a major advantage when compared to any formal data model. When a concept requires further specification, it can be combined with other existing concepts (eg, body site and device) or extended by new project-specific properties.

### Implementation Challenges

The process of clinical data acquisition passes through numerous filters before it ends up in a data warehouse for further usage. From acquisition of the data through questionnaires, formularies, texts, devices, etc in many different systems to the warehouse, several ETL processes usually will be required, resulting in loss of information. Therefore, the granularity and precision of the back-office semantic linkage can only represent the information richness known at that time. For example, the status “covid positive” cannot be coded in LOINC as this would require knowing the analytical method used by the laboratory. During that process, similar data in the data warehouse might originate from different contexts, which are not represented in the data warehouse. This is true within a care provider organization and is amplified when aggregating data originating from different care facilities and sources. These challenges were addressed in the strategy in several manners. The semantic framework with clear definitions of the concepts and their encoding in KR limited the ambiguity when creating the ETL procedures in the hospital. Second, the task of mapping the raw data to SPHN concepts was performed in each hospital by people knowing the internal data acquisition processes. Finally, the possibility to include relevant KR depending on the use case allowed the inclusion of relevant classifications used directly in care facilities, such as clinical, logistic, and billing classifications.

### Resource Challenges

The creation, evolution, and management of these semantic descriptions raise several challenges, notably scalability and coherence. Since the data sets rely on multiple external standards, there is versioning required, especially because the data considered can cover decades. The same is true for the maintenance of KR created in the project and for the infrastructure and human resources that will handle the storage and transport layers in hospitals. Most hospitals did not know RDF before the SPHN strategy. Competencies had to be built internally to ensure the sustainability of local solutions. Adoption by care facilities has thus been a critical factor to improve successful and sustainable implementation, with development of strategies for internal added value.

### Competencies and Educational Challenges

The introduction of several new approaches in care facilities (semantic-centered data handling, formal descriptive language for storage and transport, and relegating data models to the end of the data pipeline) has been a huge challenge and still encounters resistances in the information technology (IT) community. Dedicated efforts in building several working groups for semantics, RDF, and data model bridging involving numerous hospital representatives have been important to handle this challenge. This was managed by the DCC, which gathered representatives from all stakeholders. The task of identifying the list of variables to be exchanged and their prioritization was given to the research projects.

The semantic framework is bound to evolve as the user base grows, and this evolution must follow the needs of projects without compromising the strategy. This will only be possible if the strategy is well understood both centrally and at the hospital level by specialists in medical informatics within IT departments. A strong effort is therefore currently underway within the SPHN to disseminate the strategy via the publication of strategic papers, webinars, and courses given to members of the SPHN community [50-52].

### Conclusions

The main contribution of this work involves a new strategy for enabling nationwide intercommunity health data interoperability. The proposed strategy relies on the development of a semantic-based framework, which is designed to not replace existing standards but use them in a synergistic, pragmatic, and purpose-specific way. As the framework is built on the compositionality principle, it offers high flexibility and sustainability. The use of formal descriptive languages, such as RDF, as a data storage and transport layer ensures strong scalability to new needs. At the final stage, building specific bridges to fulfill the many data models used in research or required to comply with regulatory frameworks has proven successful and has been an important asset to ensure continuity of existing processes.

The wide adoption of the proposed strategy by every university hospital and high research organization in Switzerland as the national standard for sharing clinical data marks an important transition to an interoperable landscape for personalized health in Switzerland.

## Abbreviations

ATC: Anatomical Therapeutic Chemical
CDISC: Clinical Data Interchange Standards Consortium
DCC: Data Coordination Center
EHR: electronic health record
ETL: extract, transform, and load
GTIN: Global Trade Item Number
HL7: Health Level 7
i2b2: Informatics for Integrating Biology and the Bedside
ICD: International Classification of Diseases
IT: information technology
KR: knowledge representation
LOINC: Logical Observation Identifiers Names and Codes
NSB: National Steering Board
OMOP: Observational Medical Outcomes Partnership
RDF: resource description framework
RIM: Reference Information Model
SNOMED CT: Systematized Nomenclature of Medicine Clinical Terms
SPHN: Swiss Personalized Health Network
